# Debating Pros and Cons of Total Neoadjuvant Therapy in Rectal Cancer

**DOI:** 10.3390/cancers13246361

**Published:** 2021-12-18

**Authors:** Francesco Sclafani, Claudia Corrò, Thibaud Koessler

**Affiliations:** 1 Department of Medical Oncology, Institut Jules Bordet, Rue Meylemeersch 90, 1070 Anderlecht, Belgium; francesco.sclafani@bordet.be; 2 Université Libre de Bruxelles (ULB), Route de Lennik 808, 1070 Brussels, Belgium; 3 Translational Research Center in Onco-Hematology, Department of Medicine, Faculty of Medicine, University of Geneva, 1205 Geneva, Switzerland; Claudia.Corro@unige.ch; 4 Swiss Cancer Center Léman, Geneva and Lausanne, 1005 Lausanne, Switzerland; 5 Department of Oncology, Geneva University Hospital, 1205 Geneva, Switzerland

**Keywords:** total neoadjuvant therapy, rectal cancer, chemoradiotherapy, short-course radiotherapy, induction chemotherapy, consolidation chemotherapy, RAPIDO, PRODIGE-23

## Abstract

**Simple Summary:**

Rectal cancers represent one third of all colorectal tumours. Patients diagnosed with localised colon cancer undergo surgery upfront, likely followed by adjuvant chemotherapy. Those diagnosed with localised rectal cancer, however, frequently benefit from neoadjuvant treatments with either radiotherapy or chemoradiotherapy before undergoing surgery. On the other hand, the benefit of adjuvant chemotherapy in this setting is more controversial. The main challenges in treating patients affected by rectal cancer encompass: decreasing the risks of local relapse and distant metastases, preserving the sphincter and minimising treatment-associated functional sequelae, and improving overall survival. Some of these fuelled the concept of total neoadjuvant therapy, namely giving all available treatments including radiotherapy and systemic chemotherapy before surgery. Here, we critically review the pros and cons of such a treatment strategy, but also discuss the biological rational to support neoadjuvant treatment intensification.

**Abstract:**

Recently, two large, randomised phase III clinical trials of total neoadjuvant therapy (TNT) in locally advanced rectal cancer were published (RAPIDO and PRODIGE 23). These two trials compared short-course radiotherapy (SCRT) followed by chemotherapy with standard chemoradiotherapy (CRT) and chemotherapy followed by CRT with standard CRT, respectively. They showed improvement in some of the outcomes such as distant recurrence and pathological complete response (pCR). No improvement, however, was observed in local disease control or the de-escalation of surgical procedures. Although it seems lawful to integrate TNT within the treatment algorithm of localised stage II and III rectal cancer, many questions remain unanswered, including which are the optimal criteria to identify patients who are most likely to benefit from this intensive treatment. Instead of providing a sterile summary of trial results, we put these in perspective in a pros and cons manner. Moreover, we discuss some biological aspects of rectal cancer, which may provide some insights into the current decision-making process, and represent the basis for the future development of alternative, more effective treatment strategies.

## 1. Introduction

**Figure 1:**
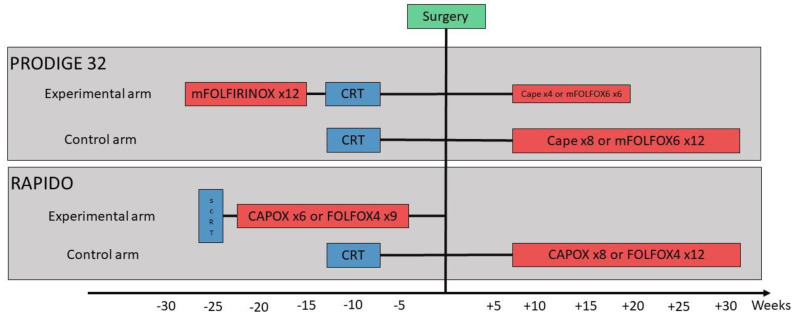
Study design of the PRODIGE 23 and RAPIDO trials.

Stage II-III rectal cancers are generally treated with neoadjuvant long-course chemoradiotherapy (CRT) or short-course radiotherapy (SCRT) before surgery, which were shown to improve local control. Two trials compared these two treatment strategies, and they are considered to be equivalent [1,2]. European and American guidelines consider CRT and SCRT interchangeable, leaning towards CRT when tumour downsizing is needed to achieve clear resection margins [3,4]. Despite these treatments, 25–30% of patients will develop distant metastases, with a high risk of ultimately dying of their recurrent disease. Indeed, the impact of adjuvant chemotherapy in this setting is strongly limited by the reduced compliance and limited, if any, effectiveness [5]. These considerations fuelled the idea to intensify the neoadjuvant treatment with systemic chemotherapy, ultimately leading to a novel approach called Total Neoadjuvant Treatment (TNT). Up until now, the literature about TNT was crowed of retrospective series and small randomised trials [6]. These early reports showed important proportions (up to 28%) of patients achieving pathological complete response (pCR), opening a window of opportunity for a watch and wait strategy for those achieving clinical complete response (cCR) [5,7]. A few months ago, the first two large, randomised phase III trials comparing TNT with the standard approach, in both cases CRT, released their preliminary results [8,9]. RAPIDO compared SCRT followed by 6 cycles of CAPOX or 9 cycles of FOLFOX4 consolidation chemotherapy and surgery (without adjuvant treatment) with CRT followed surgery and 8 cycles of CAPOX or 12 cycles of FOLFOX4 adjuvant chemotherapy, Figure 1 [8]. PRODIGE-23 compared 12 cycles of modified FOLFIRINOX followed by CRT, surgery and 4 cycles of capecitabine or 6 cycles of modified FOLFOX6 adjuvant chemotherapy with CRT, followed by surgery and 8 cycles of capecitabine or 12 cycles of modified FOLFOX6 adjuvant chemotherapy [9]. Both studies met their respective primary endpoints with statistically significant hazard ratios. Undoubtedly, they will change the practice and quickly make their way into international guidelines.

Driven by these positive results, some would argue that every eligible patient should benefit from TNT, while others would have some concerns about the routine implementation of such a practice, or even the superiority of TNT over standard neoadjuvant therapy. Several systematic reviews and meta-analyses were conducted exploring the potential benefit of TNT. All those published before 2020 used mainly phase II trials and the Polish 2 trial, reporting improvement in pCR and disease-free survival (DFS) which match overall the PRODIGE-23 and RAPIDO results. On the other hand, they also claim a survival benefit which was not found yet in the two recently published phase three trials. [10,11].

In this article, two medical oncologists expose their point of view, either in favour or against TNT, and a biologist discusses some biological aspects of rectal cancer that could help the interpretation of recent data and optimisation of management decisions.

## 2. For Total Neoadjuvant Therapy (the Point of View of FS)

Until recently, my standard approach to rectal cancer patients requiring neoadjuvant treatment included either long-course chemoradiotherapy (CRT) or short-course radiotherapy (SCRT) [12,13,14]. I have been quite critical of many colleagues who, encouraged by questionable recommendations from international guidelines, were routinely proposing total neoadjuvant therapy (TNT) to these patients [3,4]. Like many, I myself was attracted by the idea of delivering systemic chemotherapy pre-operatively, and conducted research work in this setting [15,16]. Nevertheless, I always warned my peers about the lack of convincing high-quality evidence to support the implementation of TNT into everyday practice. Since summer last year though, this evidence finally exists, consisting of two large, well-conducted, randomised phase III trials, which I strongly believe would be equally criticisable not to consider as practice-changing [8,9]. As often happens, however, data interpretation is not unequivocal, and the optimal neoadjuvant treatment for locally advanced rectal cancer (LARC) remains a matter of debate, with not everybody agreeing that RAPIDO and PRODIGE-23 have set a new standard of care.

As a strong advocate of TNT, the first point that I would like to make (a point that I feel may go unnoticed to many) is about the historical evidence-building process for nonmetastatic rectal cancer. While the last few decades have seen a substantial evolution of the treatment algorithm for this disease, none of the pivotal, superior randomised phase III trials from the post-total mesorectal excision (TME) era (with the only exception of the CAO/ARO/AIO-04 study, the results of which were not confirmed by other similar trials and did not ultimately change clinical practice) [17,18,19,20,21,22] met its primary statistical hypothesis. Over time, better local tumour control and reduced toxicity were upgraded as key secondary outcome measures to justify the adoption of new treatment strategies, whereas no associated survival improvement was ever formally demonstrated [12,13]. In this regard, the impact of RAPIDO and PRODIGE-23 is profound, breaking away from such a leit-motiv, and representing the very first trials showing an improvement in meaningful time-to-event primary outcome measures such as 3-year DFS and 3-year disease-related treatment failure (DRTF), Table 1. Some people may argue that the evidence that TNT improves overall survival (OS) is still lacking, but we should acknowledge that neither triasl was adequately powered for this endpoint, and data are still immature at this stage. Some recent data about surrogacy for rectal cancer, and the positive OS trend reported by the PRODIGE-23 investigators, are anyway encouraging [9,23].

The second point that should persuade skeptics about the value of TNT is the strong consistency of results between RAPIDO and PRODIGE-23. The risk of type I error is certainly possible with randomised controlled trials. To account for this, in evidence-based medicine, the availability of consistent high-quality studies increases confidence in the evidence, and allows for upgrading the recommendation for clinical practice to the highest level. While RAPIDO and PRODIGE-23 were conceptually similar (both addressing the value of TNT against standard neoadjuvant therapy), they had a different design, especially in relation to the investigational treatment (i.e., type, timing, and duration of neoadjuvant systemic chemotherapy, and type of radiotherapy regimen) [8,9]. Nonetheless, such differences affected neither the relative nor the absolute performance of the TNT arm, with similar hazard ratios for the primary endpoints, and identical rates of pCR being reported. Furthermore, the success of the investigational strategy of either trial was mostly attributable to the risk reduction of distant metastases, which represented the main cause of cancer recurrence after the routine adoption of total mesorectal excision (TME), and was not adequately addressed by traditional approaches [24].

The third point, which I believe is the most controversial among oncologists and generally used as the main argument against TNT, is the magnitude of the clinical benefit associated with this strategy. Many think that an absolute 7% improvement in either DFS or DRTF at 3 years is marginal, the routine use of TNT carrying an unacceptable risk of over-treatment. I suppose that this is a fair concern, as 14 patients need to be treated with TNT (instead of CRT or SCRT) to rescue only one of them from suffering a recurrence/death event. It should be pointed out, however, that the risk of over-treatment is inherent to any precautional treatment that is delivered in the peri-operative setting, and the absolute advantage of TNT as demonstrated by RAPIDO and PRODIGE-23 is higher than that of adjuvant chemotherapy for stage II colon cancer, and in line with that of peri-operative chemotherapy for liver-resected stage IV colorectal cancer, two largely endorsed and routinely adopted treatment options [25,26,27]. Most importantly, it should not be forgotten that, even if none of the so far conducted randomised phase III trials showed a whatsoever benefit for adjuvant chemotherapy following pre-operative (chemo)radiotherapy and TME surgery [28,29,30,31,32], this treatment is still included in international guidelines and frequently administered in clinical practice [3,4]. Delivering systemic chemotherapy before instead of after surgery not only improves oncological outcomes, but also allows us to spare patients from unnecessary toxicities deriving from ineffective post-operative therapies. In this regard, it is also well established that the same chemotherapy is much better tolerated when delivered pre- rather than post-operatively [33].

The fourth point, more investigational but not less relevant, is about the potential of TNT to allow adaptive, response-dependent, treatment strategies. In both RAPIDO and PRODIGE-23, the rate of pCR in the investigational arm was twice as high as in the control arm [8,9]. In practical terms, this means that the proportion of patients who could potentially become suitable for a watch and wait approach (a practice that is increasingly supported by robust albeit non-randomised evidence) [7] may be substantially higher, especially bearing in mind the broader group of patients with a complete or near complete clinical response. In support of this contention, the OPRA trial recently showed that approximately 1 out of 2 TNT-treated, stage II-III rectal cancer patients could be proposed for a non-operative management, with high chances of organ-preservation in the long term [34]. Another positive impact that the adoption of TNT (at least when given according to the PRODIGE-23 schedule) could have on patients’ quality of life may derive from the adaptive use of sequential radiotherapy. Further to the long-term sequaelae of pelvic radiotherapy [35,36,37,38,39], there is a strong interest in the investigation of radiotherapy-free approaches, which were recently showed to be feasible and not detrimental in terms of survival outcome at least in some selected cases [22,40]. A strategy of intensifying neoadjuvant therapy by delivering upfront systemic chemotherapy may actually provide an opportunity window for the re-assessment of the individual patient risk, and paradoxically lead to an overall treatment de-escalation, whereby sequential radiotherapy is offered only to non-chemotherapy-responding patients [15,41]. Of course, the feasibility of such a practice still needs to be confirmed, but, should preliminary findings be confirmed, this is something that could further tip the balance in favour of TNT in the near future.

While I strongly believe that the results of RAPIDO and PRODIGE-23 justify a paradigm shift in the management of LARC, I also appreciate that implementation of TNT into daily practice is not so straightforward. As often happens when new evidence emerges, controversy remains among physicians about how best to translate the trial findings into standard treatment recommendations, especially in relation to the optimal criteria for patient and treatment selection. In the specific case of TNT, this controversy is emphasised by key differences in study design between the two trials, which also resulted into a paradoxical mismatch between the anticipated average risk of recurrence of the patient population (higher for RAPIDO, lower for PRODIGE-23) and the intensity of the TNT strategy (higher for PRODIGE-23, lower for RAPIDO). For instance, a question that still remains unanswered is about the interchangeability of the study conclusions, that is, whether the results of RAPIDO may apply to an average-risk, PRODIGE-23-like LARC population, and whether those of PRODIGE may hold true for high-risk, RAPIDO-like LARC patients. New data will possibly become available in the future to settle such questions. In the meanwhile, however, I think that the highly consistent results from these trials should be interpreted as reproducible evidence, supporting the contention that a strategy of delivering systemic chemotherapy before surgery (according to the timing and modality of either study) is superior to conventional, neoadjuvant therapy. Two recently published, phase II studies, CAO/ARO/AIO-12 and OPRA, shade some light on the most effective sequence, with consolidation being more effective [34,42] Using a pragmatic approach, and based on the eligibility criteria and characteristics of the patients ultimately enrolled in either trial, it is reasonable to propose TNT to fit (ECOG/WHO PS <2) young (≤75 years) patients, with high-risk stage II, or any-risk stage III, tumours. Instead, the decision to adopt one TNT regimen over the other may depend on a number of factors which I previously discussed elsewhere [6]. Additional questions, which should be the subject of future investigation, include the optimal duration of consolidation chemotherapy for the RAPIDO approach, and the actual need for triplet induction chemotherapy or adjuvant chemotherapy for the PRODIGE-23 strategy. Moreover, better prognostic and predictive biomarkers are urgently needed to restrict the use of TNT to those patients who are most likely to benefit, ultimately reducing the risk of under-/over-treatment.

In conclusion, after many years of failures, two randomised phase III trials finally report on consistently positive results, supporting a change in practice for LARC. These results cannot be ignored and should prompt physicians and multidisciplinary teams to endorse TNT rapidly as a new standard of care, at least for certain patient categories.

## 3. Against Total Neoadjuvant Therapy (the Point of View of TK)

I was always a great believer in neoadjuvant treatment; treating micro-metastasis, downsizing tumours to improve surgical outcome, de-escalating the extent of surgery therefore possibly improving quality of life, testing the tumour biology, and lastly being unable to administer adjuvant chemotherapy; all these were good reasons for me to promote the neoadjuvant approach and naturally the TNT approach. In June 2020, during the virtual ASCO meeting, the presentation of the PRODIGE 23 and RAPIDO trials was amongst the most important with potential immediate change of practices; none of them relied on new drugs. In preparation of the possible positive outcomes, I conferred with our lead radio-oncologist and lower gastrointestinal surgeon to be able to deploy these new approaches the day after. However, what disappointing results I obtained instead!

We should first recognise that there exists a paucity in the definition of what is TNT. The “total” might be understood as receiving ALL oncological treatments (SCRT or CRT and chemotherapy) before surgery as in the RAPIDO trial [8]; others see it as receiving SOME additional chemotherapy with the standard of care (SCRT or CRT) before undergoing surgery followed by compulsory adjuvant chemotherapy as in the PRODIGE-23 trial [9] or left to the discretion of the investigators as in the Polish II study [43,44]. This point might seem futile to some, but it might help others to avoid mixing RAPIDO and PRODIDGE-23 treatment scheduled in their daily practice, as the sequence might be important to reach the potential benefit attached to this approach.

The most two prominent trials in the TNT setting (RAPIDO and PRODIGE-23) were recently published [8,9]. Both used a rather similar primary endpoint, namely disease-free survival (DFS) at 3 years. In PRODIGE-23, it is defined as the time from randomisation until the first cancer-related event defined as local or metastatic recurrence, second cancer, or death from any cause. The RAPIDO trial used a disease-related treatment failure at 3 years, defined as the first occurrence of locoregional failure, distant metastasis, a new primary colorectal tumour, or treatment-related death. Both trials showed an improvement in their primary outcome with a statically significant decrease from 30.4% to 23.7% (P: 0.019) for RAPIDO and a statically significant increase from 69% to 76% (P: 0.034) in the PRODIGE-23 trial, Table 1. Both trials also reported a decrease in distant metastases’ relapse of 7% in PRODIGE-23 and 6.8% in RAPIDO compared to CRT, corresponding exactly to the magnitude of the benefit seen in their primary endpoint. None of the trials showed benefits on locoregional relapse. This clearly highlights the fact that more chemotherapy before surgery will only diminish the risk for distant spread and has no impact on local control.

Everybody can appreciate that decreasing the risk of metastasis is interesting; however, this does not translate into an overall survival benefit. In RAPIDO, the 3-year overall survival is 89.1% (95% CI 86.3–92.0) in the TNT arm and 88.8% (95% CI 85.9–91.7) in the standard of care arm (P: 0.59), and, in PRODIGE-23, the 3-year overall survival rate is 91% (95% CI 86–94) in the TNT group and 88% (95% CI 83–91) in the standard of care group [8,9]. Some will argue that data are still immature, but with a median follow up of 46.5 months (PRODIGE-23) and 56 months (RAPIDO), it is very unlikely that a clinically meaningful difference will be seen in the future. 

The third point is that safety and toxicity are issues with TNT. In PRODIGE-23, 7% of patients had a grade 4—life threatening—adverse event (AE) during the neoadjuvant FOLFIRINOX part before CRT. The most common AE was neutropenia (5%) with GCSF administered in 26% of the patients. Furthermore, one patient died of a treatment-related event during the neoadjuvant chemotherapy part, which is unacceptable in a curative setting. The percentage of a grade 4 AE during CRT is similar in both arms, as is the number of grade 5: one per arm. Preoperative CRT was administered in 95% of patients in the TNT arm and 99% of the standard arm in PRODIGE-23 (P: 0.019). Standard fractionated radiotherapy was delivered as planned in similar proportions of patients in both groups. In RAPIDO, 6.5% of patients experienced a grade 4 AE in the experimental arm versus 2.3% in the standard of care arm during the perioperative therapy time with one grade 5 treatment related in each arm before surgery. This excess toxicity during neoadjuvant treatment did not have an impact in either trial, on the number of patients having surgery (92% TNT group versus 89% in the standard of care arm, P: 0.086 in RAPIDO and 92% TNT group versus 95% in the standard of care arm, P: 0.26 in PRODIGE-23). Three times more patients undergoing TNT should expect to develop a grade 4—life threatening—AE during TNT compared to the standard of care. Not only is TNT more toxic and therefore not to be offered to frail patients or those with comorbidities, but the medical team should be prepared to respond swiftly to these life-threatening situations.

My fourth point is, although we do not currently have the final data for the quality of life (QOL) in either trials, data presented during the annual ASCO meeting in 2020 showed no significant benefit in RAPIDO in the overall health, overall quality of life, or low anterior resection syndrome (LARS) at three years, and those included in the PODIGE-23 publication show no significant benefit in overall global health status nor QOL scores either [9]. It might be reassuring to some, however, as one of the goals of TNT is the early size reduction in large symptomatic tumours; it seems that one more expected benefit is not there.

Lastly and most importantly, TNT did not change the type of surgery that was performed nor the percentage of colostomy needed. PRODIGE-23 showed no difference in terms of the surgery performed (P: 0.303); most patients had low anterior resection (78.9% in TNT arm versus 74.4% in control arm) followed by abdominoperineal resection (14.1% versus 14.0%) and intersphincteric resection in either arm (7.0% versus 10.7%) [9]. The same is observed within RAPIDO (P: 0.56) with most patients having low anterior resection (758% in TNT arm versus 55% in control arm) followed by abdominoperineal resection (34% versus 39%) [45]. Despite this, a doubling in pCR with the TNT strategy has no impact on the type of surgery performed. Furthermore, neither trial demonstrated an impact of this intensification on the percentage of patients requiring temporary (78.4% in experimental arm versus 77.8% in control arm) or permanent (14.1% versus 14.6%) stoma, as demonstrated in PRODGE-23 [9]. This last point is of the greatest importance as “what patients really want” is not achieved with the TNT strategy. Indeed, Harrison and colleagues showed that 63% of patients will give up a mean of 34% of their life expectancy to avoid having a stoma [46]. In fact, when confronted with different outcomes within one case description, patients and clinicians disagree on the order of importance of the outcomes. If they both agree that life expectancy is the most important outcome, patients value avoiding surgery with permanent stoma more and worry less about recurrence. Interestingly, patients find the duration of disease-free survival the least important of the outcomes. [47].

In conclusion, have RAPIDO and PRODIGE-23 changed practice? Yes, certainly! However, with no benefit on local control, or surgery de-escalation, and currently no overall survival benefit or quality-of-life benefit, they do match what is important for patients and as such should not be profusely and mindlessly used.

## 4. Effect of Chemotherapy and Chemo-Radiotherapy in the TME (Claudia Corro)

The tumour microenvironment (TME) is established by a complex network of interactions between epithelial cells, immune cells, endothelial cells, and stromal fibroblasts [48]. In addition to the cellular components, biochemical and biophysical cues modulate cell behaviours and shape tumour evolution [49]. Notably, cancer treatments can directly affect the cellular and extracellular components of the microenvironment [50]. While the majority of chemotherapeutic agents carry out their anti-cancer effects through the disruption of DNA replication and transcription machinery [51,52], the primary mechanism of action of radiotherapy (RT) is DNA damage through a radiation beam. Increasing evidence suggests that, in addition to the direct killing of tumour cells, chemotherapy and RT can modulate the tumour milieu and enhance anti-tumour immunity. The cell death caused by these agents can act as a trigger for the recruitment of lymphoid and myeloid cells [49,53]. This is potentially caused by the release of tumour-specific antigens (neoantigens) and proinflammatory signals from the dying cells, the alteration of the tumour vasculature, and the trafficking of immune cells to the tumour site [54,55,56].

Several studies investigated the dynamic changes in the immune cell composition of TME before and after treatment in rectal cancer (RC) with the aim to identify markers predictive of response [57,58,59]. For instance, the expression of a few well-known T cell markers (CD3, CD8, CD45RO, and FoxP3) were evaluated in matched-pair tumour biopsies before and after FOFOX chemotherapy in 8 locally advanced RC patients [60]. This study reported a reduction in FoxP3+ Tregs that was associated with a favourable response to therapy [60]. Similarly, in another study, a decrease in FoxP3+ TILs correlated with better therapeutic responses to FOLFOX chemotherapy alone or in combination with RT (25–50 Gy) [61]. Remarkably, this effect was not only limited to the tumour site. In fact, CRT was also shown to induce systemic immune changes such as a reduction in circulating Tregs and myeloid-derived suppressor cells (MDSCs) [62]. Consistent with a previous report, RC patients treated with CRT displayed higher numbers of CD4+/CD8+ cells in the TME after CRT [63,64]. A significant increase in CD3+ and CD8+ immune infiltrates in the TME of RC patients after neoadjuvant CRT was prognostic for the extent of tumour regression, distant metastasis rates, and DFS [65]. In contrast, a multivariate analysis revealed that, while the total number of CD3+ and CD8+ TILs was significantly lower in RC after neoadjuvant CRT compared to primarily resected cases, the level of GZMB+ CD8+ T cells was increased and positively correlated with tumour regression and lower recurrence [66]. Taken together, these data demonstrate that chemotherapy and CRT can induce an immune-active microenvironment by recruiting immune cells into the tumour site, and that this phenomenon is correlated with improved clinical outcomes.

Nonetheless, it is worth mentioning that the immune infiltrate may have variable effects depending on the cell type, activation state, and spatial organisation [67]. For instance, a few studies highlighted how certain immune cells (i.e., FoxP3+ Tregs [68,69], macrophages [70,71,72,73,74], dendritic cells [75,76,77]) may have both positive and negative associations with patient outcomes. Furthermore, a detailed resolution analysis of the immune cell composition within the CRC TME revealed a localisation-dependent prognostic relevance of T cell densities, highlighting the importance of interaction networks [78]. Alongside high levels of CD8+ TILs, the expression of immune checkpoint genes such as B7-H3 and B7-H5 was found upregulated upon CRT in RC patients [79]. Similarly, a high expression of PD-1, PD-L1, and MHC-I in tumour nests was associated with neoadjuvant chemotherapy and CRT treatment [60,61,80,81]. These results may suggest that the upregulation of immune checkpoint molecules could result in a negative feedback mechanism that reduces T cell function and causes immune tolerance.

In addition to the remodelling of the TME, one general drawback of CRT treatment is the development of mechanisms of acquired resistance that may impair therapy efficacy. For instance, the hypoxic and acidic TME as a consequence of CRT is responsible for slowing down cell cycling, down-regulating DNA damage responses, reducing apoptosis, and increasing autophagy properties [82]. A series of in vitro experiments in CRC cell lines showed that low oxygen levels result in a decreased expression of pro-apoptotic proteins and contribute to resistance to oxaliplatin [83]. Another study reported the association between aerobic glycolysis and 5-FU resistance [84]. Altogether, these events lead to both genotypic and phenotypic heterogeneity. Indeed, CRT may enhance cell plasticity, genetic instability, and epigenetic reprograming. Acquired resistance to 5-FU was observed in vitro upon long-term exposure of a CRC cell line to 5-FU through the upregulation of multidrug-resistant mechanisms and stem-cell-like properties [85]. In addition, chemotherapy was found implicated in ADAM17 activation and, consequently, acquired mechanisms of resistance [86]. Taken together, these data indicate that the complex environmental changes following therapy have the potential to influence the evolution of the tumour, ultimately affecting tumour progression and clinical outcomes.

Recent evidence suggests that TNT may improve patient outcomes in RC by enhancing DFS and reducing metastatic spread [8,9]. This effect may be mediated by the cumulative immunomodulatory activity of these treatments on the TME, for instance, by further reducing immunosuppressive cells (MDSCs, immunosuppressive/M2 macrophages, and Tregs) while concomitantly increasing the infiltration of pro-inflammatory CD4+ and CD8+ T cells. At the same time, induction chemotherapy or CRT may provide the tumour adequate time to regress, enabling patients to recover from acute radiation toxicities and improving surgical procedures. Although PRODIGE-23 and RAPIDO studies highlighted TNT as an appealing option for RC patients, concerns remain about the potential risk that induction chemotherapy may drive accelerated cell repopulation and the development of resistant clones, which could reduce the effect of TNT. These concerns are reflected by the unsatisfactory local disease control and lack of OS benefit in patients receiving TNT [6,87]. It is important to mention that inter- and intra-patient heterogeneity is indeed a considerable hurdle in determining therapy responses between and within cancer patients. Remarkably, no pre-clinical or translational study evaluating the dynamic changes in the composition of the RC TME before and after TNT has been conducted so far. Similarly, the analysis of biological fluids (liquid biopsies) could inform predictive molecular biomarkers. Therefore, more efforts should be directed towards pre-clinical research to understand how TNT translates into specific phenotypes at the functional level. Moreover, future clinical trials associated with a robust translational program will shed light on the mechanistic principles of TNT as well as help identify the molecular features that predict benefits to TNT and support clinical decision making.

After years in which the practice of TNT has largely been based on assumptions and suboptimal evidence, RAPIDO and PRODIGE-23 finally provided the medical community with robust data and useful insights about the added value of pre-operative systemic chemotherapy when delivered either before or after standard radiotherapy. These two well-designed trials changed the practice, and guidelines will change accordingly. With more neoadjuvant treatment options available, clinicians will have to face the challenge of weighing the benefit of each one of those against the individual patient/tumour risk profile, always bearing in mind patients’ goals, preferences, and expectations. It is clear that one-size-fits-all approaches should never be pursued in routine practice, but the pros and cons of any management decision should always be carefully considered and shared with patients. Of course, tumour biology should ideally have a central role in the decision making, but neither for TNT nor for any other alternative treatment option is there validated predicted biomarkers which are ready for prime time. We hope comprehensive correlative analyses from ongoing and future trials will help clarify the place of TNT within the therapeutic algorithm of locally advanced rectal cancer and contribute to the implementation of truly personalised treatment strategies in this setting.

## Figures and Tables

**Table 1:** Safety and efficacy data from PRODIGE 23 and RAPIDO trials.

Outcome	Prodige 32		Rapido	
	Experimental Arm	Control Arm	Experimental Arm	Control Arm
	disease-free survival		disease-related treatment failure	
Primary endpoint	75.5%	68.5%	30.4%	23.7%
	HR: 0.69; 95% CI 0.49–0.97; *p* = 0.034		HR: 0.75; 95% CI 0.60–0.95; *p* = 0.019	
pCR	27.8%	12.1%	28.0%	14.0%
	*p* < 0.001		OR: 2.37; 95% CI 1.67–3.37; *p* < 0.0001	
Locoregional Failure (rate at 3 years)	4.0%	6.0%	8.3%	6.0%
	HR: 0.78; 95% CI 0.34–1.79; *p* = 0.56		HR: 1.42; 95% CI 0.91–2.12; *p* = 0.12	
Metastasis-free survival (rate at 3 years)	79.0%	72.0%	20.0%	26.8%
	HR: 0.64; 95% CI 0.44–0.93; *p* = 0.017		HR: 0.69; 95% CI 0.54–0.90; *p* = 0.0048	
Overall survival (at 3 years)	90.8%	87.7%	89.1%	88.8%
	HR: 0.64; 95% CI 0.44–0.93; *p* = 0.017		HR: 0.69; 95% CI 0.54–0.90; *p* = 0.0048	
Ro resection	95%	94%	90%	90%
	*p* = 0.63		*p* = 0.87	
Compliance to (chemo)radiotherapy	98%	98%	100%	93%
Compliance to neoadjuvant chemotherapy	92%	75% (adj chemo)	85%	67% (adj chemo)
Grade ≥ 3 AEs during neoadjuvant therapy	46.8% (during chemo)		48%	25%
	37.2% (during chemorad)	35.6% (during chemorad)		
Post-operative complications	29%	31%	50%	47%
Treatment related death	4%	5%	3%	3%

HR: Hasard ratio, pCR: pathological complete response.
